# Higher extracellular volume is associated with longer bypass times at corrective surgery and reduced exercise tolerance in children late after repair of tetralogy of Fallot

**DOI:** 10.1186/1532-429X-18-S1-Q25

**Published:** 2016-01-27

**Authors:** Eugenie Riesenkampff, Wietske Luining, Mike Seed, Shi-Joon Yoo, Cedric Manlhiot, Brian W McCrindle, Lars Grosse-Wortmann

**Affiliations:** grid.42327.300000000404739646Hospital for Sick Children, Toronto, ON Canada

## Background

Unfavorable left ventricular (LV) remodelling is associated with adverse prognosis after tetralogy of Fallot (TOF) repair. The aim of this study was to measure the extracellular volume (ECV) as a marker of diffuse LV myocardial fibrosis in children after TOF repair, and to understand its etiology and clinical significance.

## Methods

In this prospective, cross-sectional study ECV as a quantitative marker of diffuse fibrosis was measured in the LV myocardium using a modified look-locker inversion recovery (MOLLI) sequence. ECV was related to bypass and cross-clamp times at the time of surgery, to ventricular and myocardial function as well as to exercise tolerance.

## Results

There was no difference in ECV between 31 TOF patients (23.6 ± 3.3, age at CMR 13.9 ± 2.4 years, 19 male) and 15 controls (23.4 ± 3.0, age at CMR 13.4 ± 2.6 years, 7 male). ECV correlated with z-scores of LV end-diastolic volumes and of right ventricular (RV) end-diastolic and end-systolic volumes (r = 0.39, p < 0.05; r = 0.46, p < 0.01; and r = 0.43, p < 0.05, respectively). LV ECV did not correlate with with LV or RV ejection fraction, indexed right and left ventricular enddiastolic volumes or pulmonary regurgitation fraction or regurgitant volumes. Female TOF patients had higher ECVs as compared to males (25.7 ± 1.8% versus 22.9 ± 3.1%, p < 0.005). There were no gender differences in controls. Bypass-time during complete repair correlated with LV ECV (r = 0.46, p < 0.05). Patients who had undergone either a valve sparing repair or received a valved right ventricle to pulmonary artery conduit had a lower ECV than those after transannular patch repair (22.8 ± 2.7% versus 26.0 ± 3.0%, p < 0.05).Maximum workload on exercise testing, as a percent of predicted based on reference populations, correlated inversely with ECV (r = -0.62, p < 0.05). There were no correlations between ECV and myocardial strain, strain rate or torsion.

## Conclusions

Although children after TOF repair did not present with higher ECV than controls in this pilot study the amount of diffuse myocardial fibrosis was associated with longer bypass-times at the time of initial repair. There is early evidence that increased fibrosis is associated with decreased exercise tolerance in young patients after TOF repair. Female patients appear to have more diffuse myocardial fibrosis than males consistent with their generally less favourable hemodynamics after TOF repair.

